# Structural *versus* Functionally-informed Supratotal Resection: Where do we stand?

**DOI:** 10.1016/j.bas.2025.104398

**Published:** 2025-08-28

**Authors:** Yasir A. Chowdhury, Richard Gullan, Keyoumars Ashkan, Francesco Vergani, Ranjeev Bhangoo, José Pedro Lavrador

**Affiliations:** a Department of Neurosurgery, King's College Hospital Foundation Trust, London, UK; b Catolica Medical School, Oeiras, Portugal

To the Editor,

There is mounting evidence that supratotal resection (SupTR) of IDH-wildtype glioblastoma and grade 4 IDH-mutant astrocytoma yield superior outcomes in progression-free survival (PFS) and overall survival (OS) compared to gross total resection (GTR) (Verly et al., 2025). We would like to highlight how the definition and practice of SupTR must be integrated with functional preservation in this cohort of patients.

Whilst there is an accepted method to assess GTR, the challenge for us is now to standardize a definition for SupTR to enable a more rigorous comparison of PFS and OS in GTR versus SupTR. A purely volumetric or anatomical definition of SupTR, such as a RANO Class 1 supra-maximal contrast-enhancing resection, must be balanced with a *functional* definition beyond radiological tumor. A personalized surgical strategy is even more crucial in the setting of the SupTR to maintain a successful onco-functional balance.

To undertake a *maximum safe resection*, we would like to highlight the temporal triad of neuro-oncology surgery: (1) pre-operative functional anatomy assessment, (2) intra-operative clinical, electrophysiological and fluorescent guidance and (3) post-operative interventional neurorehabilitation.

Pre-operative assessments focus on cortical functional and subcortical structural mapping. Functional magnetic resonance imaging (fMRI) and navigated transcranial magnetic stimulation (nTMS) are established modalities for motor and language and mapping (Leone et al., 2025). Recent developments in nTMS have pursued higher cognitive and functional assessments such as mapping of arithemetic calculation and the supplementary motor area (Bonaudo et al., 2024). Functional-based tractography, such as nTMS or fMRI seeded tracts, has revolutionized pre-operative decision-making of patients’ onco-functional balance. Furthermore, it enhances the pre-operative preparation of the intra-operative strategy. Overall, pre-operative mapping (POM) data combined with intra-operative neuromonitoring (IONM) leads greater GTR with reduced post-operative neurological deficits compared to standalone utilization of IONM (Baig et al., 2025).

The effective utilization of pre- and intra-operative techniques to preserve function is even more essential in the context of SupTR where resection margins beyond contrast enhancement are inevitably going to encroach functional anatomy. The intra-operative use of 5-aminolevulinic acid (5-ALA) already facilitates GTR of high-grade gliomas and is frequently a surrogate marker for disease beyond radiological contrast enhancement. A connectome-based resection, whereby intra-operative awake clinical assessment of higher order functions (Lavrador et al., 2025), allows a maximum safe functional SupTR and provides a standardized taxonomy in the procedure. Furthermore, a connectome-based approach to high grade glioma resection allows for neuroplasticity to develop which will be crucial for an increased quality-of-life orientated PFS and OS in this cohort of patients (Price et al., 2024).

Patients undergoing SupTR close to functional boundaries are more likely to develop transient post-operative deficits. Both postoperative nTMS mapping and tractography have been reported as valuable tools to predict motor recovery after surgery (Seidel et al., 2019). Patients with high-grade gliomas cannot afford a significant delay to adjuvant therapy and thus SupTR would normally be contraindicated in near-eloquent tumors. In our center, we now use nTMS as a tool to potentiate recovery in patients who develop post-operative paresis (Rosenstock et al., 2025). Incorporating this interventional neuro-rehabilitation allows faster recovery following surgery, decreases surgery to chemoradiotherapy time and increases extent of resection (EoR) (see Fig. 1).

**Fig. 1:**
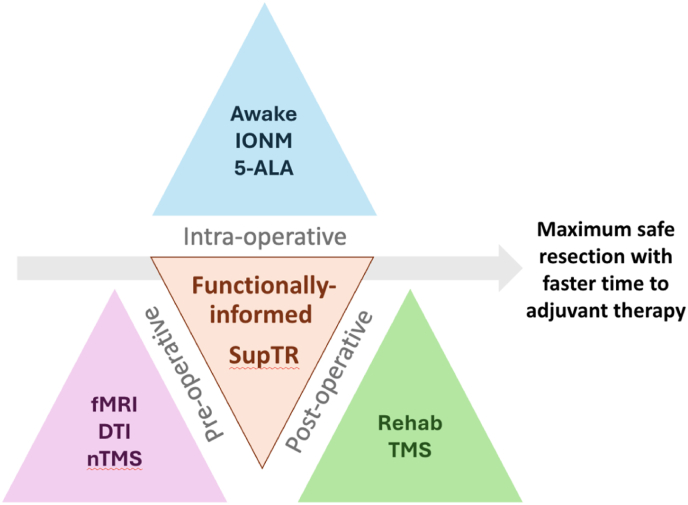
The temporal triad of functionally-informed SupTR.

As we continue to explore the boundaries of surgical intervention in malignant gliomas, it is imperative that future systematic reviews and meta-analyses distinguish between a structural SupTR and functionally-informed SupTR (fSupTR). The latter represents the future of high-grade glioma surgery: precision-guided, patient-specific, and ethically grounded in the preservation of quality of life. The success and standardization of a maximum safe functional resection will be a great challenge and will be influenced by surgical center experience and the application of constantly evolving knowledge of the human connectome. There will be a need to study the quality of life and long-term outcomes in patients with malignant gliomas undergoing GTR versus SupTR versus fSupTR.

In conclusion, we urge the field to now take the next step: integrate functional preservation as a central tenet of supratotal resection within the pre-, intra- and post-operative care of patients with high-grade gliomas.

## Author contributions

YAC: Conceptualization, writing and editing. RG: Reviewing. KA: Reviewing. FV: Reviewing. RB: Reviewing. JPL: Conceptualization and reviewing.

## Ethical adherence

The production of this publication required no ethical board review.

## Funding & Conflicts of interest

The authors declare no financial support was received for the research and/or publication of this article. No authors have any conflicts of interest to declare.
